# The usefulness of dual channel elastomeric pump for intravenous patient-controlled analgesia in geriatrics: a randomized, double-blind, prospective study

**DOI:** 10.1186/s12871-022-01733-2

**Published:** 2022-07-07

**Authors:** Chung Hun Lee, Soo Ah Cho, Seok Kyeong Oh, Sang Sik Choi, Myoung Hoon Kong, Young Sung Kim

**Affiliations:** Department of Anesthesiology and Pain Medicine, Korea University Guro Hospital, 148, Gurodong-ro, Guro-gu, Seoul, 08308 Korea

**Keywords:** Dual chamber device, Elastomeric pump, Geriatrics, Patient-controlled analgesia, Variable-rate feedback

## Abstract

**Background:**

Intravenous patient-controlled analgesia (IV-PCA) is often used in the postoperative period. However, determining an appropriate opioid dose is difficult. A previous study suggested the usefulness of variable-rate feedback infusion. In this study, we used a dual-channel elastomeric infusion pump to provide changes in PCA infusion rate by pain feedback.

**Methods:**

Ninety patients undergoing orthopedic surgery of American Society of Anesthesiologists grade I-III and 65 to 79 years of age participated in the study. All patients were given a dual-chamber PCA. Patients were randomly allocated to a treatment group (Group D; PCA drugs divided into both chambers) or control group (Group C; PCA drugs only in the constant flow chamber with normal saline in the adjustable flow chamber). The primary outcome was the amount of fentanyl consumption via PCA bolus. The secondary outcome variables were pain score, total fentanyl consumption, rescue analgesic use, patient satisfaction, recovery scores, and adverse events including postoperative nausea and vomiting (PONV).

**Results:**

Group D showed decreased fentanyl consumption of the PCA bolus, a decrease in rescue analgesic use, and better patient satisfaction compared with group C. The incidence of PONV was much higher in group C. There was no difference in other adverse events.

**Conclusions:**

We showed the usefulness of dual chamber IV-PCA to change the flow rate related to pain feedback without any complications. Our results suggest a noble system that might improve existing IV-PCA equipment.

**Trial registration:**

The study registered at UMIN clinical trial registry (registered date: 05/03/2020, registration number: UMIN000039702).

**Supplementary Information:**

The online version contains supplementary material available at 10.1186/s12871-022-01733-2.

## Background

Bolus injection and continuous infusion are two methods of intravenous (IV) drug administration. As many clinicians know, both methods have limitations from the viewpoint of pharmacokinetics. If the target opioid plasma concentration is provided, a sufficient bolus dose shows an exponential decay with a transiently higher plasma concentration, followed by a concentration lower than the target level [1]. Therefore, a single bolus dose of opioid may cause complications in the early phase and insufficient pain control in the later phase. In contrast, a continuous infusion maintains a consistent plasma concentration. However, there is a delay before reaching that level [1], which means difficulty in early pain control.

Among numerous pain control methods, opioid-based intravenous patient-controlled analgesia (IV-PCA) is commonly used to relieve postoperative pain [2, 3]. IV-PCA can be provided by the two methods described above, which make up for the shortcomings of each method, which is appropriate and plausible. However, problems still occur in clinical situations. A basal infusion dose that is too low may cause pain and require more opioid bolus use whereas a dose that is too high may cause PCA cessation. Moreover, the optimal opioid concentration is not constant because many factors including pain level, and intraindividual and interindividual differences are involved [4].

Many PCA devices have been developed and used since the 1960s [5]. Initially, most types of PCA were electronic, but currently, non-electronic, elastomeric types are widely used. Compared to electronic types, elastomeric types have several advantages including being light and easy to carry, disposable, simple to manipulate, not requiring a power source, and no program error [6, 7]. However, most non-electric IV-PCA devices have a single elastomeric pump, which makes it difficult to change the preset rate. We observed a phenomenon in which the pain score was high on the first day after surgery indicating a shortage of analgesic dose whereas the pain score decreased on the second day with increased frequency of opioid complications. We assumed that pain management would be optimized by increasing the analgesic dose on the first day and decreasing the dose on the second day according to the pain level of each patient.

The Bellomic®M (Cebika, Uiwang-si, Gyeonggi-do, Republic of Korea) is a newly designed IV-PCA device that consists of a dual infusion elastomeric pump with two balloon chambers. One channel contains a continuous flow-rate chamber and has a bolus function, whereas the other channel contains an adjustable flow-rate chamber without a bolus function.

The purpose of this study was to use this dual channel elastomeric IV-PCA for elderly patients undergoing orthopaedic surgery and evaluate the efficacy of dual-channel IV-PCA compared to single-channel IV-PCA and single placebo channel via PCA opioid consumption, postoperative pain, rescue analgesic, subjective satisfaction of the patient, and other adverse events. We hypothesized that more efficient pain management would be achieved if the flow rates were adjusted according to patient pain feedback using this dual-channel IV-PCA device.

## Material and methods

### Design

This study was a single-centre prospective randomized controlled trial conducted from 2019 to 2020. We followed the Consolidated Standards of Reporting Trials (CONSORT) guidelines when designing this study. After receiving approval from the institutional review board, it was registered at the UMIN clinical trial registry (registered date: 05/03/2020, registration number: UMIN000039702).

Elderly patients 65–79 years of age, of American Society of Anesthesiologists (ASA) class I-III, undergoing orthopaedic surgery, excluding patients with a body mass index (BMI) > 35, drug abuse or dependence, history of drug allergy (to fentanyl, ramosetron, nefopam, and other nonsteroidal anti-inflammatory drugs), cognitive disorder, dementia, other psychiatric disease, speech impairment, and severe chronic pain other than at the surgical site, were included. Written informed consents were obtained from all the subjects. Demographic data including age, sex, weight, height, ASA class, and underlying diseases including hypertension, diabetes mellitus, and cerebrovascular accident were collected from all the patients.

All patients received a dual-chamber PCA just after arriving in the post-anaesthesia care unit (PACU). Patients were randomly allocated to a treatment group (Group D; PCA drugs divided into both chambers) or control group (Group C; PCA drugs only in the constant flow chamber with normal saline in the adjustable flow chamber), and they were unaware of the group assignment. One independent investigator was responsible for the generation of the random allocation sequence and the group assignment of patients. Randomization was done in two blocks using a web-based computer-generated list (www.randomization.com). The subject numbers, which were kept in opaque sealed envelopes, were checked only by an independent anaesthesiologist who did not have a role other than making PCA in the operating room. The patients and other clinical investigators were blinded to the group allocation.

The primary outcome in this study was the amount of fentanyl consumption via PCA bolus. A power analysis proposed that a sample size of at least 40 patients for each group would be required with a significance level of 5% to achieve 80% power. Effect size was calculated from the PCA consumption (volume delivered at 12–24 h) reported in a previous study [8], which compared the constant-rate and variable-rate infusion. To allow for an exclusion rate, the study population was prospectively set at 90 patients.

### IV-PCA device and regimen

The Bellomic®M (Cebika, Uiwang-si, Gyeonggi-do, Republic of Korea) is a dual channel infusion elastomeric pump. The basal-bolus channel provides a 2 mL/h fixed flow rate infusion and a bolus function (1 ml of the bolus volume, 10 min lock-out period). The other selector channel can be set to 0, 1, or 2 ml/h with an adjustable flow rate without a bolus function. The maximum volume of each chamber is 100 ml.

**Fig. 1:**
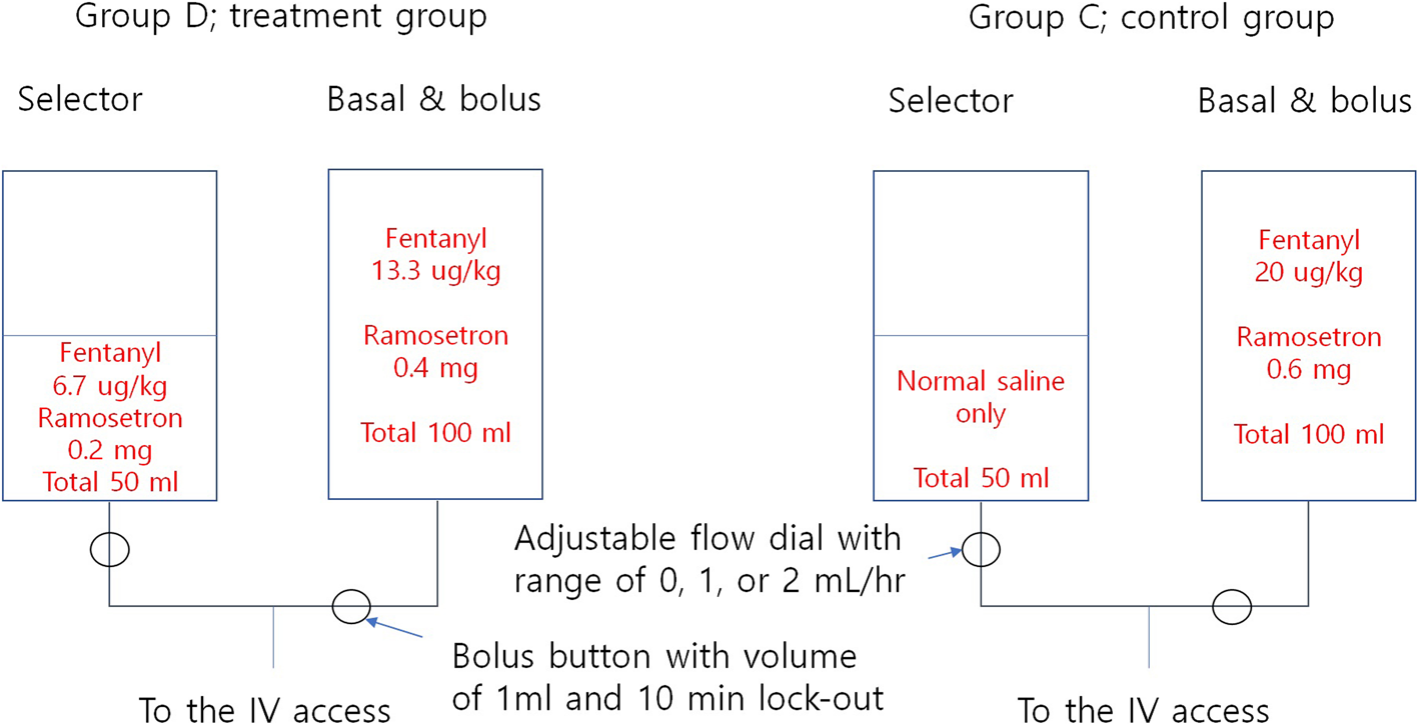
A schematic of the dual chamber IV-PCA regimen. This figure shows the composition of the dual chamber IV-PCA device and our regimen. The IV-PCA consists of two channels: a basal-bolus channel with a fixed flow rate infusion and a bolus function, and the other selector channel has an adjustable flow rate without bolus function. Group D: PCA drugs were divided into both chambers. Group C: PCA drugs were contained only in the constant flow chamber and normal saline contained in the adjustable flow chamber

In group D, the IV-PCA contained fentanyl 13.3 μg/kg + ramosetron 0.4 mg with normal saline to a total volume of 100 ml in the basal-bolus channel with a constant rate of 2 ml/h and fentanyl 6.7 μg/kg + ramosetron 0.2 mg with normal saline to a total volume of 50 ml in the selector channel with adjustable rates of 0–2 ml/h. In group C, IV-PCA contained fentanyl 20 μg/kg + ramosetron 0.6 mg with normal saline to a total volume of 100 ml in the basal-bolus channel with a constant rate of 2 ml/h and 50 ml of normal saline in the selector channel with adjustable rates of 0–2 ml/h. In both groups, the initial rate of the selector channel was 1. When the pain score (visual analogue scale, VAS) was 7 or more in the PACU and 5 or more in the ward, the flow rate of the selector channel was increased by 1 (maximum 2), and when the pain score was 3 or less, the rate was decreased by 1 (minimum 0) (see the perioperative management in the method section). When the flow rate was fixed at 1 without adjustment, except for an additional bolus dose, the amount and infusion rate of fentanyl used in group D and group C became the same. A schematic of the IV-PCA regimen is shown in Fig. 1.

### Perioperative management

All patients were monitored by pulse oximetry, electrocardiography, automatic noninvasive blood pressure measurement, bispectral index (BIS), capnography, end-tidal concentration of volatile anaesthetics, and train-of-four (TOF). Blood pressure was monitored at 5-min intervals and the heart rate was monitored continuously. After preoxygenation, anaesthesia was induced with an intravenous 2 mg/kg of propofol bolus by rocuronium 0.6 mg/kg. After loss of consciousness, tracheal intubation was performed. Patients received 0.6–2 vol% of sevoflurane with remifentanil infusion for the maintenance of anaesthesia. The depth of anaesthesia was adjusted during surgery to maintain BIS values of 40 to 60 and mean blood pressures at 80 to 120% of the baseline value. Minute ventilations were adjusted to maintain normocapnia with an FiO_2_ of 0.5, and 3 l/min of fresh gas flow rate during surgery. Initial settings of tidal volume were 8 ml/kg, I:E of 1:2, and airway pressure that was adjusted to not exceed 25 cmH_2_O. At the end of surgery, all anaesthetics were stopped, and ventilator settings were changed to FiO_2_ 1.0 and 8 l/min of fresh gas flow rate. Then, 2 mg/kg of sugammadex was administered. Return of neuromuscular function was confirmed using TOF peripheral nerve stimulation. Before extubation, four equal twitches in the TOF were required. Extubation was performed when a patient was awake (defined as spontaneous eye opening or purposeful movements to verbal commands).

IV-PCA was initiated just after arrival to the PACU. A simple mask with an oxygen flow rate of 5 l/min was applied to patients in the PACU. The pain score, level of consciousness, and modified Aldrete post-anaesthesia recovery score (PARS) were evaluated every 5 min. The pain score was evaluated using the VAS with a range from 0–10 points. When the pain score was 7 or more, the flow rate of the selector channel was increased by 1 (maximum 2) and fentanyl 1 μg/kg was intravenously administered as a rescue analgesic. Rescue analgesic can be administered up to two times (maximum fentanyl 2 μg/kg with at least a 15-min interval) in the PACU. When the pain score was 3 or less, the flow rate of the selector channel was decreased by 1 (minimum 0). The Richmond Agitation and Sedation Scale (RASS) and the Confusion Assessment Method for the Intensive Care Unit (CAM-ICU) were used to determine the level of consciousness and assess delirium. Any adverse events including postoperative nausea/vomiting (PONV), dizziness, dry mouth, episode of bronchospasm, laryngospasm, or oxygen desaturation (SpO_2_ < 95%) were recorded. Then, 0.075 mg of palonosetron was administered to patients with PONV. If there was no abnormality, the patients stayed in the recovery room for 60 min and were then moved to the ward.

At 6, 12, 24, 36, and 48 h after arriving at the PACU, pain scores and fentanyl consumption in PCA were evaluated. In addition, the patient’s subjective satisfaction and any side effects including nausea/vomiting, dizziness, dry mouth, respiratory depression, hypotension, and other complications were recorded. Patient satisfaction was assessed using a 5-point rating scale (1 = very dissatisfied, 2 = dissatisfied, 3 = neutral, 4 = satisfied, 5 = very satisfied). When the pain score was 5 or higher in the ward, the flow rate of the selector channel was increased by 1 (maximum 2), and intravenous nefopam 20 mg was administered as a rescue analgesic over 15 min.

### Statistical methods

After confirming normality by the Kolmogorov–Smirnov test, data expressed as the mean ± SD were compared between the two groups using independent t-tests or the Mann–Whitney *U*-test where appropriate. Data expressed as the number of patients were compared using chi-square analysis when any cells with expected values below 5 did not exceed 20% in the contingency table or Fisher’s exact test where appropriate. Repeated measures analyses of variance (RM-ANOVA) were applied for repeated measured variables including fentanyl consumption in PCA and pain score for the analysis of group effects. We adopted the results of tests showing a within-subject effect when the sphericity condition of data was satisfied or the results from multivariate analyses.

Statistical analyses were performed using the Statistical Package for Social Sciences, version 22.0. *P*-values below 0.05 were considered statistically significant.

## Results

A total of 87 patients were enrolled in this study excluding 3 patients who withdrew consent (43 patients for group D and 44 patients for group C). There was no significant difference in the demographic data between the two groups (Table 1). Total anaesthesia time, operation time, fluid input and output, recovery profiles including PARS, RASS, CAM-ICU, and fentanyl consumption in PACU were not different between the two groups. The rescue analgesics in the ward were required more often in group C (Table 2).

**Table 1:** Demographic data

	Group D (*n* = 43)	Group C (*n* = 44)	*p*-value
Age (years)	70.93 ± 3.81	70.39 ± 3.90	0.512
Sex (M/F)	17 / 26	16 / 28	0.761
Weight (kg)	62.59 ± 11.91	63.96 ± 10.78	0.574
Height (cm)	158.34 ± 8.55	160.16 ± 8.40	0.319
ASA class (II/III)	35 / 8	39 / 5	0.344
Hypertension (Y/N)	28 / 15	26 / 18	0.563
DM (Y/N)	11 / 32	10 / 34	0.756
CVA (Y/N)	6 / 37	3 / 41	0.196

Values are either the mean ± SD or the number of patients. Group D: PCA drugs were divided into both chambers. Group C: PCA drugs were contained only in the constant flow chamber, but normal saline was contained in the adjustable flow chamber. There was no significant difference between the two groups

*ASA class* American Society of Anesthesiologists physical status classification, *DM* Diabetes Mellitus, *CVA* Cerebrovascular Accident

**Table 2:** Perioperative outcomes and recovery profiles

	Group D (*n* = 43)	Group C (*n* = 44)	*p*-value
Anesthetic time (min)	152.44 ± 63.34	169.09 ± 90.85	0.324
Operation time (min)	104.49 ± 55.60	117.02 ± 81.43	0.403
Transfusion (Y/N)	0 / 43	1 / 43	1.000
Colloid use (Y/N)	0 / 43	1 / 43	1.000
Total fluid (mL)	741.63 ± 381.31	902.16 ± 638.94	0.158
Blood loss (mL)	118.14 ± 84.30	156.82 ± 152.82	0.147
Urine output (mL)	200.81 ± 207.26	251.93 ± 314.15	0.374
PARS in PACU at 1 h	9.91 ± 0.29	9.89 ± 0.32	0.756
RASS in PACU at 1 h	-0.09 ± 0.29	-0.09 ± 0.29	0.973
CAM-ICU in PACU at 1 h (positive/negative)	4 / 39	4 / 40	1.000
Fentanyl bolus in PACU (mg)	56.51 ± 48.62	69.77 ± 54.25	0.234
The number of fentanyl bolus in PACU	0.95 ± 0.79	1.11 ± 0.84	0.362
Nefopam as rescue analgesics in the ward (mg)	17.67 ± 23.99^*^	29.55 ± 30.95	0.049
Patient satisfaction on POD 1	4.14 ± 0.83	4.02 ± 1.00	0.556
Patient satisfaction on POD 2	4.44 ± 0.70^*^	4.00 ± 0.99	0.019

Values are mean ± SD or number of patients. ^*^p < 0.05 compared to the group C. Group D: PCA drugs were divided into both chambers. Group C: PCA drugs were contained only in the constant flow chamber, but normal saline was contained in the adjustable flow chamber. There was no significant difference between the two groups. *PARS* modified Aldrete Post-anesthesia Recovery Score, *PACU* Post-anesthesia Care Unit, *RASS* Richmond Agitation and Sedation Scale, *CAM-ICU* Confusion Assessment Method for the ICU, *POD* Postoperative Day

**Fig. 2:**
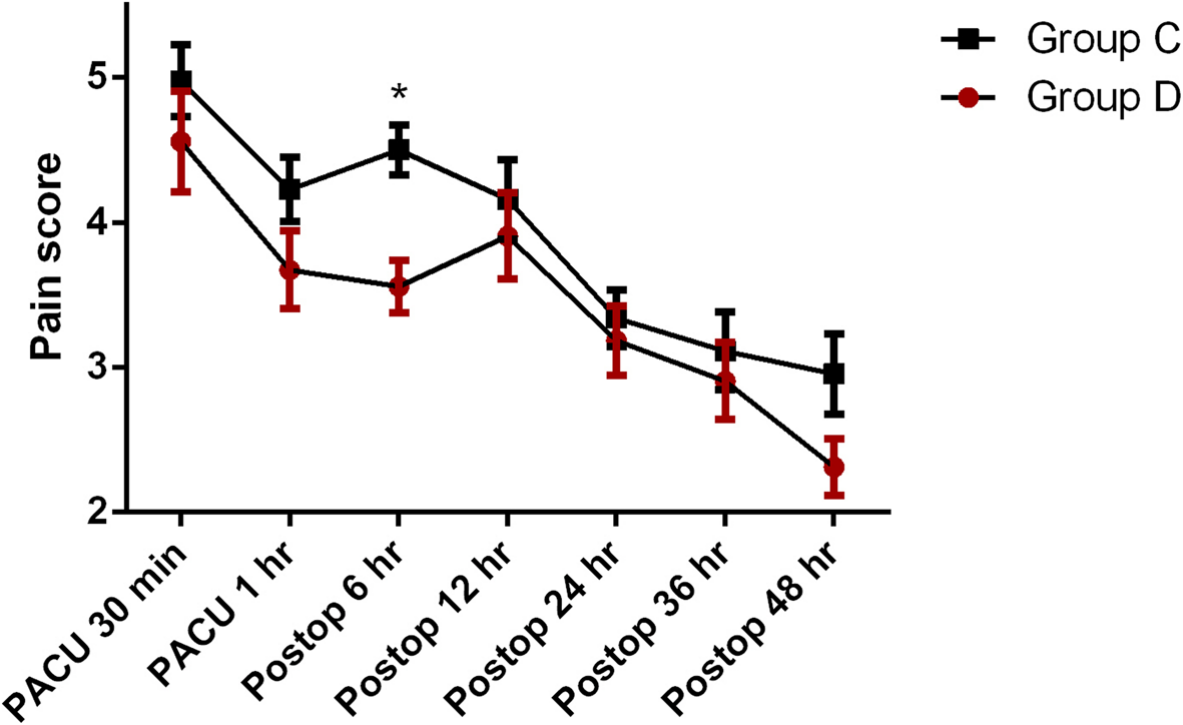
Changes in pain scores in the postoperative periods. There were no significant differences in the pain scores observed between the two groups (*p* = 0.081, multivariate analysis). At 6 h after arrival in the PACU, the pain score in group D was lower than that in group C (*p* < 0.001). Group D: PCA drugs were divided into both chambers. Group C: PCA drugs were contained only in the constant flow chamber with normal saline contained in the adjustable flow chamber. The plot is represented by ‘mean with SEM’ instead of ‘mean with SD’ for visibility (See Table 3)

**Fig. 3:**
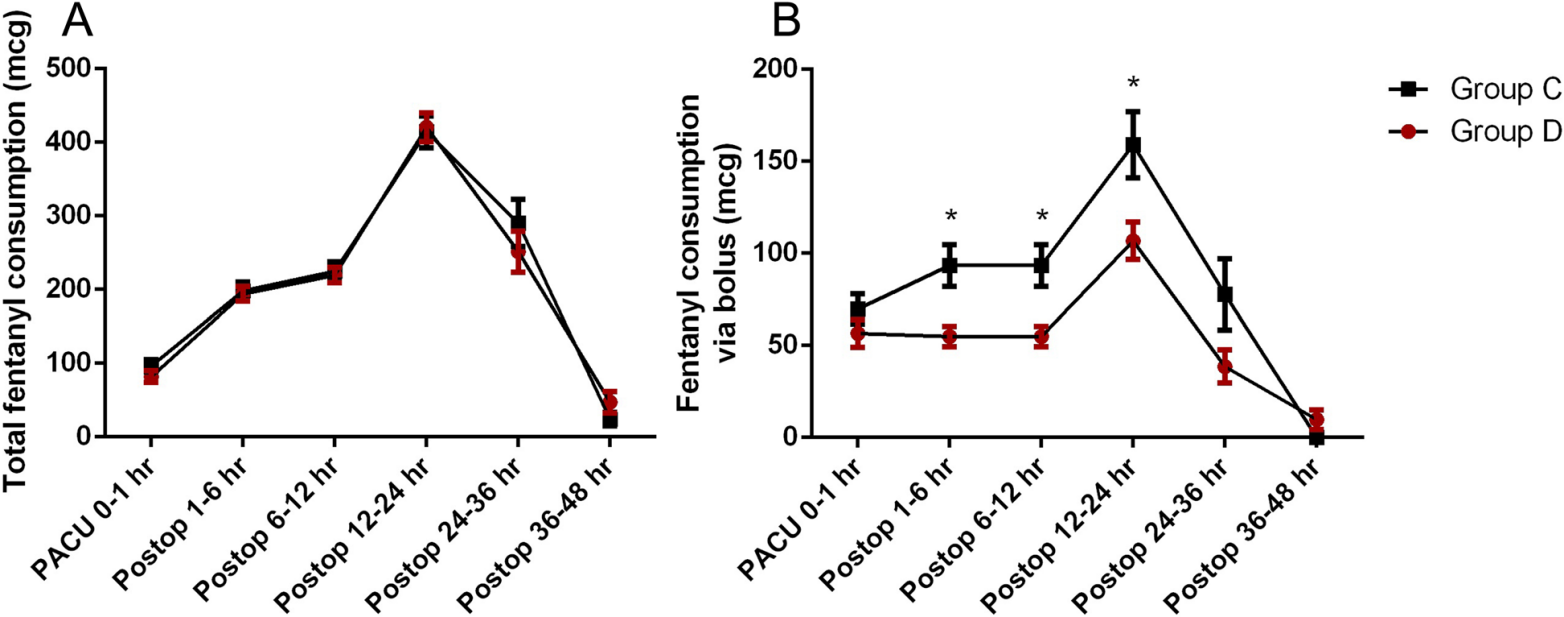
Changes in fentanyl consumption in the postoperative periods. **A** Total fentanyl consumption. There were no differences in total fentanyl consumption (*p* = 0.315, multivariate analysis). **B** Fentanyl consumption via bolus. Fentanyl consumption used as boluses were different between the two groups (*p* < 0.001, multivariate analysis). The amounts of fentanyl administered as bolus during postoperative 1–6, 6–12, and 12–24 h were significantly lower in group D compared to group C. Group D: PCA drugs were divided into both chambers. Group C: PCA drugs were contained only in the constant flow chamber with normal saline contained in the adjustable flow chamber. The plot is represented by ‘mean with SEM’ instead of ‘mean with SD’ for visibility

Group D showed a significantly lower pain score at 6 h after arrival in the PACU compared to group C (*p* < 0.001). However, multivariate analysis did not reveal any significant differences in the changes of pain intensity or infusion rate of selector channel between the two groups (*p* = 0.081, 0.541, respectively) (Fig. 2, Table 3). Even though there were no differences in total fentanyl consumption, the amounts of fentanyl administered as bolus were not the same at all time points between the two groups (*p* = 0.315, *p* < 0.001, respectively) (Fig. 3).

**Table 3:** Changes in infusion rate of PCA selector channel and pain score in the postoperative period

	Group D (*n* = 43)	Group C (*n* = 44)	*P*-value
Infusion rate			0.541
Post op 0.5 h	1.09 ± 0.65	1.16 ± 0.57	
Post op 1 h	1.05 ± 0.72	1.16 ± 0.61	
Post op 6 h	1.07 ± 0.70	1.14 ± 0.63	
Post op 12 h	1.05 ± 0.72	1.02 ± 0.70	
Post op 24 h	0.91 ± 0.72	0.98 ± 0.70	
Post op 36 h	0.91 ± 0.68	0.95 ± 0.71	
Pain score			0.081
Post op 0.5 h	4.56 ± 2.27	4.98 ± 1.64	
Post op 1 h	3.67 ± 1.76	4.23 ± 1.48	
Post op 6 h	3.56 ± 1.18	4.50 ± 1.15	
Post op 12 h	3.91 ± 1.94	4.16 ± 1.80	
Post op 24 h	3.19 ± 1.56	3.34 ± 1.29	
Post op 36 h	2.91 ± 1.74	3.11 ± 1.77	
Post op 48 h	2.31 ± 1.28	2.95 ± 1.83	

Values are either the mean ± SD or the number of patients. Group D: PCA drugs were divided into both chambers. Group C: PCA drugs were contained only in the constant flow chamber, but normal saline was contained in the adjustable flow chamber. There was no significant difference between the two groups

The amounts of fentanyl administered as bolus at postoperative 1–6, 6–12, and 12–24 h were much lower in group D compared to group C (Fig. 3). Patient satisfaction was much higher in group D compared to group C (Table 2).

At postoperative day 2, the PONV incidence decreased compared to operation day in group D (20.9% on operation day to 4.7% at postoperative day 2, *p* = 0.049), but not in group C (18.2% on operation day to 18.2% at postoperative day 2). There were no significant differences for other complications (Table 4).

**Table 4:** Postoperative complications

	Group D (*n* = 43)	Group C (*n* = 44)
	Operation day / Postoperative day 1 / Postoperative day 2	Operation day / Postoperative day 1 / Postoperative day 2
Nausea/vomiting	9 / 8 / 2^†^	8 / 9 / 8
Dizziness	2 / 4 / 2	2 / 4 / 5
Dry mouth	12 / 6 / 6	9 / 5 / 4
Desaturation (< 95%)	5 / 0 / 0	2 / 0 / 0
Profound hypotension	1 / 0 / 0	2 / 0 / 0
Profound hypertension	0 / 0 / 0	1 / 0 / 0
Chest discomfort	1 / 0 / 0	0 / 0 / 0
Shivering	2 / 0 / 0	0 / 0 / 0
Dysuria	0 / 0 / 1	1 / 0 / 0
Sleep apnea	1 / 0 / 0	0 / 0 / 0
Sweating	0 / 0 / 0	0 / 1 / 1
Constipation	0 / 0 / 1	0 / 0 / 0

Values are number of patients. ^†^*p* = 0.024 compared to operation day. Group D: PCA drugs were divided into both chambers. Group C: PCA drugs were contained only in the constant flow chamber, but normal saline was contained in the adjustable flow chamber

## Discussion

This study demonstrated the usefulness of dual-chamber IV-PCA with a feedback control infusion rate. There were significant decreases in the amount of fentanyl bolus and rescue analgesic with higher patient satisfaction when the dual channels of PCA were both activated.

Postoperative pain can be classified as musculoskeletal, visceral, and persistent post-surgical pain. The primary afferent nociceptors A-delta and C fibres have an important role in acute pain [9]. During acute pain, the depolarization of A-delta and C fibre nociceptors, termed transduction, develops. Nociceptive input to the dorsal horn also increases sympathetic activity in the spinal cord and sympathetic ganglia. The threshold of pain is usually stimulus-specific and reproducible in different individuals and in the same individual at different times. However, the mechanisms of postoperative pain are more complex. Repetitive nociceptive stimuli may lead to central sensitization [10]. Sensitization and primary hyperalgesia occur as a decrease in the threshold for pain. Pain tolerance is also dependent upon psychological factors as well as the stimulus itself. These make it difficult to set an endpoint for pain control.

The concept of a minimum effective analgesic concentration (MEAC) may help to establish a strategy for pain management. MEAC explains the phenomenon that pain decreases dramatically at a specific point as a result of administering the drug in small increments [11]. Considering the existence of inter- and intra-individual differences, the MEAC cannot be determined from the plasma concentration of opioids [12]. Consequently, the titration of drugs should be performed individually to obtain the MEAC. It is also necessary to maintain a constant plasma concentration while avoiding levels that are too high or low. Considering these characteristics, IV-PCA seemed better than IM injection or pro re nata (prn) bolus.

The next step was to select the drugs and dose for IV-PCA. We examined previous studies to determine the ideal IV-PCA regimen [13, 14]. In this study, we used fentanyl for IV-PCA. Because of its rapid onset and short duration of action, fentanyl is preferred over morphine for IV-PCA [4]. Shin et al. [4] reported the use of a lower background infusion rate, younger age, and absence of adjuvant analgesics were independent risk factors for rescue analgesic administration. In contrast, a higher background infusion rate, female gender, and absence of 5HT_3_ receptor blockers were independent risk factors for rescue antiemetic administration. A higher background infusion rate may increase daily opioid consumption, and consequently may increase the incidence of adverse effects. However, a higher background infusion rate did not always lead to better pain relief and improved sleep patterns [15]. Shin et al. [4] recommended a proper background infusion rate of fentanyl ranging from 0.12 to 0.67 mcg/kg/h. In our regimen, the background infusion rates of fentanyl were calculated as 0.4 mcg/kg/h for group C and 0.26, 0.4, or 0.54 mcg/kg/h for group D, which were within their recommended ranges.

Lee et al. [8] applied variable-rate feedback infusion by IV-PCA. They suggested pain management through a change in the background infusion rate according to the patient’s pain level and needs would be more efficient with lower dosages of analgesics. Their study had an important influence on the design of our study. They showed variable-rate feedback infusion provided a significant decrease in the number of bolus and volume delivered by PCA. In our study, there was also a decrease in the amount of fentanyl bolus even there was no difference in the total fentanyl consumption. Rather than pressing the bolus button because of extreme pain, we assumed that maintaining a certain level of the required drug concentration through a variable rate control in advance would have beneficial effects on pain control or patient satisfaction [16].

We also assumed that as the pain intensity steeply decreased, adverse effects including PONV and dizziness might become troublesome. To reduce PONV, we mixed ramosetron in the PCA and used prn palonosetron in the PACU after considering their pharmacokinetic properties [17]. Our results showed a PONV difference two days after the surgery. We thought an adjustment of the PCA infusion rate might have reduced unnecessary opioid infusion. In the study by Lee et al. [8], the PONV risk was reduced from 33 to 18% over the whole period. Even mild PONV may cause a longer hospital stay, lower patient satisfaction, and higher medical costs [18]. From the anaesthesiologist’s point of view, many side effects on the day after surgery may be easily overlooked. However, these side effects have a great influence on patient recovery [19].

It is natural that efficient pain control and a lack of side-effects induce higher patient satisfaction. Chumbley et al. [20] identified three factors—having better pain relief, not worrying about ‘giving oneself too much drug’, and not experiencing feeling ‘peculiar in the head’—that make the patient feel positive about PCA. To add to this explanation, dizziness, hallucinations, and nightmares are within the term ‘peculiar in the head’. Although the incidence of dizziness did not reach statistical significance between the two groups, it was clear that the satisfaction level was reduced among patients who complained of dizziness (data not shown).

We also focused on the occurrence of delirium because old age and inadequate pain control are known factors of delirium [21]. Eight cases were reported as transient positive CAM-ICU in the PACU. However, there were no cases of delirium during the follow-up period of up to 7 days after the operation. This was thought to be because most of the patients were relatively healthy and there were few cases of massive bleeding during surgery. In high-risk patients, there may be different consequences, so further studies are required.

There were other limitations. We did not consider adjuvant analgesics in the PCA although we usually use a combination of fentanyl, hydromorphone, ketorolac, or nefopam. When we designed this study, we chose the simplest regimen to avoid ambiguous interpretation. However, to fully utilize our dual channel, it is likely that additional experiments with a combination of several drugs will be required. That is, this study alone was not enough to demonstrate the full potential of the dual channel PCA pump. In addition, because we simply targeted the pain score as feedback, there was no discrimination between opioid tolerance or opioid-induced hyperalgesia. In this case, it might have been better to use another analgesic instead of opioids. We used nefopam as a rescue medication in the ward. A previous study [22] reported 20 mg of nefopam was equal to 12 mg of morphine. We could have converted 1 mg of nefopam to 6 mcg of fentanyl, but we excluded the use of nefopam when calculating the total amount of fentanyl used because of potential drug interactions [23].

Nevertheless, this study demonstrated the successful application of a new developmental form of IV-PCA. Considering few studies have used a dual chamber PCA, our regimen might help clinicians apply a similar type of PCA. With considering the simplicity of use and fewer incidence of device related-errors [24] of elastomeric pump and precision control of electronic PCA, our dual-chamber PCA may combine the strengths of both types. Our findings will have a significant impact on the development of the PCA device, which has been stagnant for many years. Efforts to improve postoperative pain control are meaningful as long as uncomfortable patients remain.

## Conclusions

In conclusion, this study demonstrated the clinical application of a new form of IV-PCA for geriatrics. Dual chamber IV-PCA by changing the flow rate to pain feedback provided efficient analgesia, with reduced PONV, and high patient satisfaction without severe complications.

## Supplementary Information

**Additional file 1.** Consort diagram.

**Additional file 2.** Specific information of the PCA device usedin the study. It contains the design, shape, function, and dimension ofdetailed components of the device.

## Data Availability

The datasets generated and analyzed in this study are available in the UMIN-ICDR Case data repository, [https://upload.umin.ac.jp/cgi-bin/icdr_e/ctr_dl_his_list.cgi?recptno=R000045281].

## Abbreviations

ASA class: American Society of Anesthesiologists physical status classification
BIS: Bispectral index
CAM-ICU: Confusion Assessment Method for the Intensive Care Unit
IV-PCA: Intravenous patient-controlled analgesia
MEAC: Minimum effective analgesic concentration
PACU: Post-anesthesia care unit
PARS: Modified Aldrete post-anesthesia recovery score
PONV: Postoperative nausea/vomiting
RASS: Richmond Agitation and Sedation Scale
RM-ANOVA: Repeated measures analyses of variance
TOF: Train-of-four
VAS: Visual analogue scale
