# Supplementary figures and images for: The usefulness of dual channel elastomeric pump for intravenous patient-controlled analgesia in geriatrics: a randomized, double-blind, prospective study
Source: BMC Anesthesiol. 2022 Jul 7;22:210. doi: 10.1186/s12871-022-01733-2 (PMC9261015; doi:10.1186/s12871-022-01733-2)

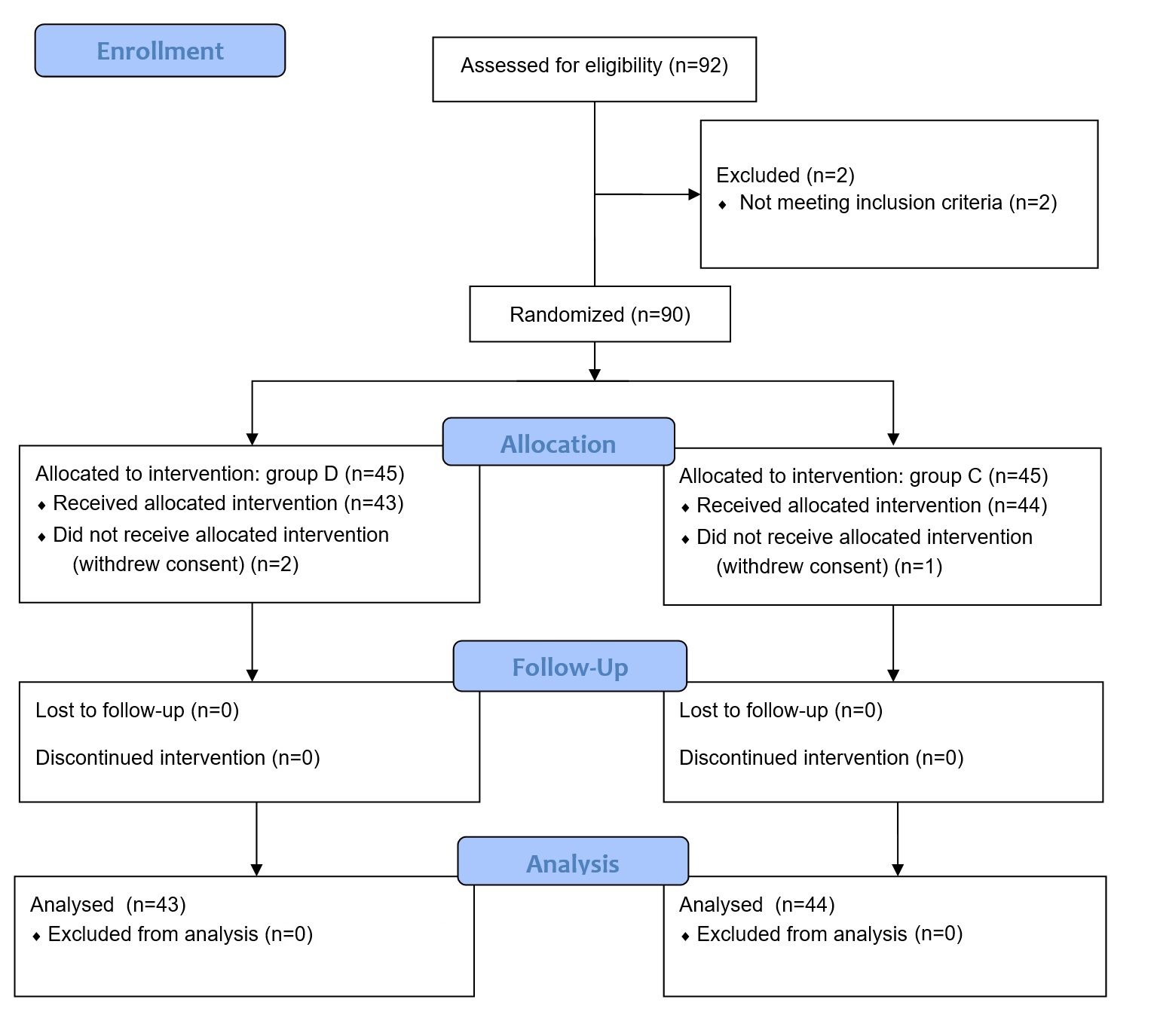

Supplement: Supplementary file 1 — Additional file 1. Consort diagram. [file 12871_2022_1733_MOESM1_ESM.jpg]
