# Supplementary material for: The usefulness of dual channel elastomeric pump for intravenous patient-controlled analgesia in geriatrics: a randomized, double-blind, prospective study
Source: BMC Anesthesiol. 2022 Jul 7;22:210. doi: 10.1186/s12871-022-01733-2 (PMC9261015; doi:10.1186/s12871-022-01733-2)
Supplement: Supplementary file 2 — Additional file 2. Specific information of the PCA device usedin the study. It contains the design, shape, function, and dimension ofdetailed components of the device. [file 12871_2022_1733_MOESM2_ESM.pdf]

Bellomic PCA specification

1) Introduction

This product is a disposable medical device as an infusion pump used to inject a certain amount of medicine into a patient. The drug solution is injected with the pressure generated in the drug storage unit with elastic force. There are products with a button for additional bolus injection and a product with a flow controller to control the amount of drug injected.

2) Product name

Bellomic® - Silicone Balloon Infusion	Bellomic® Signature (S)
	Bellomic® Compact (C)
	Bellomic® Light (L)
	Bellomic® Petite (P)

3) Product specification

(1) General specification

※ Multi Balloon Pump

Type	Specificatoion	contents
Bellomic® Petite (P)<br>- Dual Continuous Petite Type	Volume Reservoir (mL)	200 mL (100/100)
	Total Flow rate (mL/hr)	0.5/0.5, 0.59/0.59, 1/1, 2/2, 4/4, 5/5, 6/6, 8/8, 10/10
	Tube Material	PVC, PU
	Air Eliminating Filter (㎛)	1.2, 5.0

(2) Bolus specification

Specification	Content
Bolus Dose (mL)	1 for multi balloon pump
Refill Time (min)	10 for multi balloon pump

(3) Selector specification

Specification	Content
Flow rate (mL/hr)	0, 1, 2, priming

**Bellomic® Multi Balloon Pump**	
24) Dual Continuous Petite Type 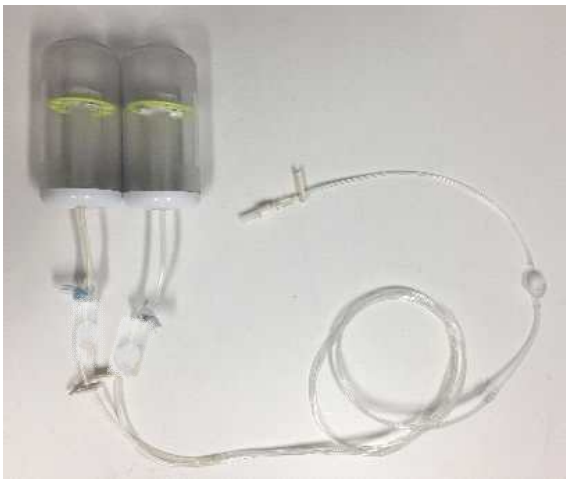	25) Triple Continuous Petite Type 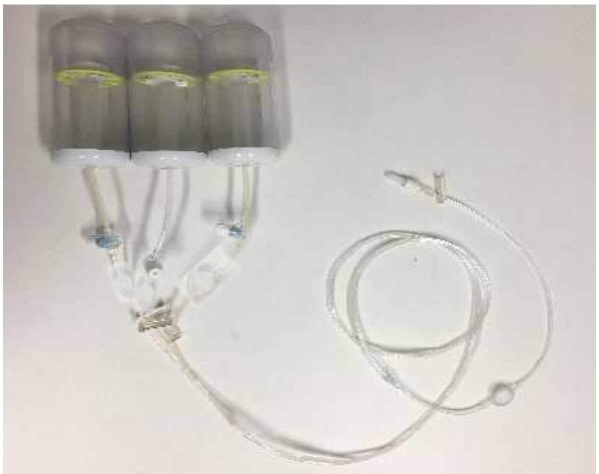

※ Accessories

1) Selector Lock device

- As a locking device for the selector, it serves to fix the set flow rate so that it cannot be adjusted.

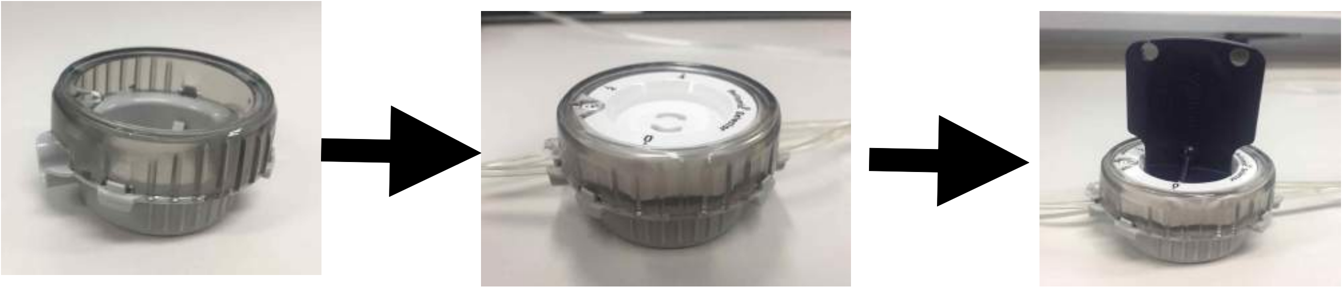

2) Bolus Lock device

- As a locking device for the bolus, it prevents the injection of additional drugs into the bolus in an unintentional situation.

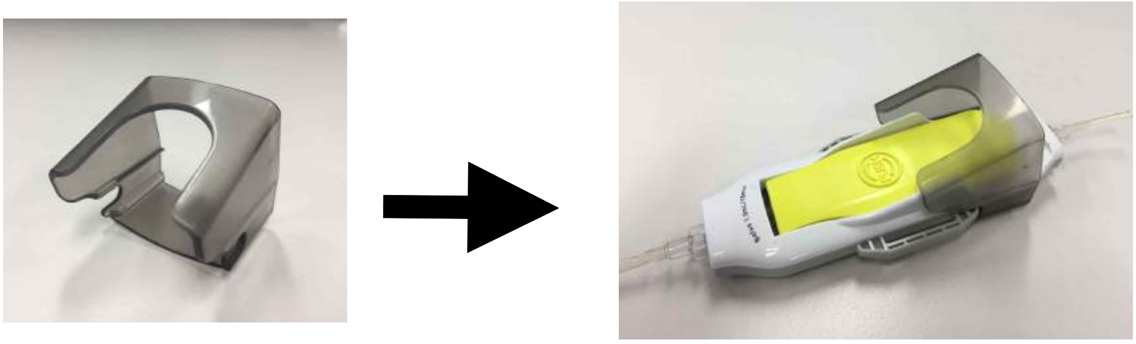

Dual Continuous Petite Type

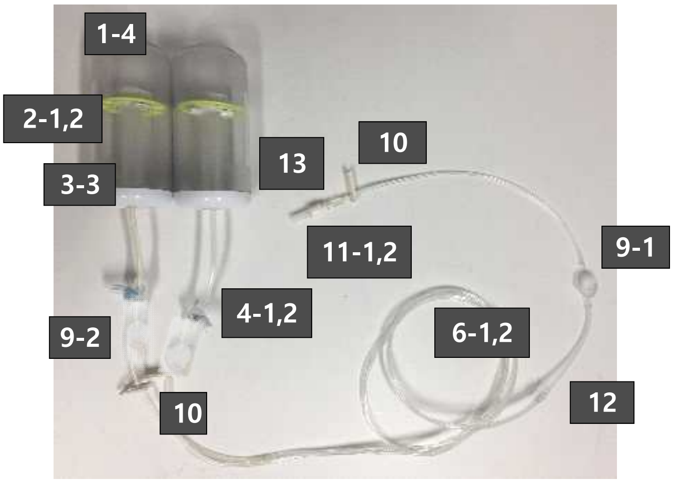

Maximal capacity	Models		
200mL (100/100mL)	MUNBS0(P)	MUNBS4(P)	MUNBS8(P)
	MUNBS1(P)	MUNBS5(P)	MUNBSX(P)
	MUNBS2(P)	MUNBS6(P)	MUNBSW(P)

Number	Name		Function
1-1	Outer housing1 (Signature)		Protects internal contents from external impacts
1-2	Outer housing2 (Compact)		
1-3	Outer housing3 (Light)		
1-4	Outer housing4 (Petite)		
2-1	Housing Silicone Guide		Displays the amounts of injected volume by printed scale
2-2	Inner housing	Supporter	Supports silicone material during expansion for drug injection
		Silicone	The parts where the drug is stored.
3-1	Bottom houising1 (Signature/Light)		Protects internal contents from external impacts
3-2	Bottom housing2 (Compact)		
3-3	Bottom housing3 (Petite)		
4-1	T Injection Port	Body	A port that used to inject the drug or be connected to other devices
4-2		Cap	Protects 'T injection port' from foreign material or contamination.
5	I-connector		A two-way connectopr that allows the drug to flow in a straight line.
6-1	Connector Tube (PVC)		Channels which the drug flows
6-2	Connector Tube (PU)		
7-1	Precision flow controller (Selector)	Case	The outside of the regulator which regulates the flow rate of the drug in various ways and protects the internal parts.
		Glass tube	Acts as a capillary tube to control the flow rate of the drug.
		Silicone Rubber	Prevents drug leakage and helps the user to regulate certain amount of drug to flow.
		Control Lever	Help the user set the flow rate of the drug.
		Pin	Device that helps the precision flow controller to be fixed.
7-2	Precision flow controller (Selector_M)	Top Case_M	Help the user set the flow rate of the drug
		Glass tube	Acts as a capillary tube to control the flow rate of the drug
		Silicone Rubber_M	Prevents drug leakage and helps the user to regulate certain amount of drug to flow.
		Body_M	The internal part that can control the flow rate by inserting a glass tube that controls the flow rate of the drug.
		Bottom Case_M	Protects internal parts and prevents leakage.
		Pin_M	Device that helps the precision flow controller to be fixed.

8-1	PCM (Bolus)	Top Case	An external component to the switch for additional drug injection; It protects the internal components.
		Push Lever	Button which is used to inject additional bolus.
		Bottom Case	Protects internal parts and prevents water leakage, combined with the 'Top Case'
		Silicone	A channel which the drug flows
		Check Valve	A part which prevents the backflow
		Keeping Chamber	A storage for bolus drugs
		Glass tube	Acts as a capillary tube to control the flow rate of the drug
8-2	PCM (Bolus_F)	Top Case_F	An external part of the button for bolus administration; it protect the internal parts.
		Silicone Button_F	A button for bolus administration
		Bottom Case_F	An external part of the button which holds the button, combined with the top case; It protects the internal parts.
		Silicone	A channel which the drug flows
		Check Valve	A part which prevents the backflow
		Keeping Chamber	A storage for bolus drugs
		Glass tube	Acts as a capillary tube to control the flow rate of the drug
9-1	Filter 1		Filter foreign substances from the drug and remove air from the connecting tube.
9-2	Filter 2		
10	Slide Clamp		A device which blocks the flow of the drug
11-1	Rotating Male Luer	Body	Combined with or separate from the Female Luer.
11-2		Cap	Protects the Rotating Male Luer from foreign substances or contamination.
12	2-Way Connector		Allows the drug to flow in one direction between the two direction
13	Restrictor Connector		Connects the glass tube to the tube

Dual Continuous Petite Type

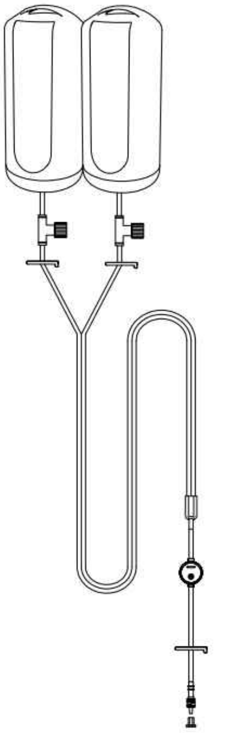

Length (± 10%)	150 cm		
Maximal capacity	Models		
200mL<br>(100/100mL)	MUNBS0(P)<br>MUNBS1(P)<br>MUNBS2(P)	MUNBS4(P)<br>MUNBS5(P)<br>MUNBS6(P)	MUNBS8(P)<br>MUNBSX(P)<br>MUNBSW(P)

※ Dimensions

<table>
<tr><th>Number</th><th colspan="2">Name</th><th>Dimensions (unit: mm, error range: ±10%)</th></tr>
<tr><td>1-1</td><td rowspan="3">Signature</td><td>Outer housing1</td><td rowspan="3">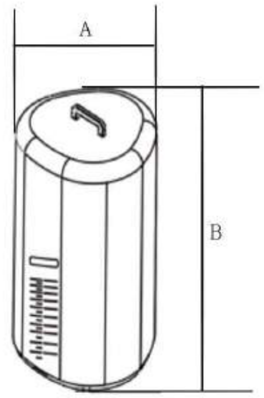

<table>
<tr><th></th><th>150 mL</th><th>300 mL</th><th>600 mL</th></tr>
<tr><td>A</td><td colspan="3">67.66</td></tr>
<tr><td>B</td><td>130.50</td><td>160.50</td><td>190.50</td></tr>
</table></td></tr>
<tr><td>2-2</td><td>Inner housing1</td></tr>
<tr><td>3-1</td><td>Bottom housing1</td></tr>
<tr><td>1-2</td><td rowspan="3">Compact</td><td>Outer housing2</td><td rowspan="3">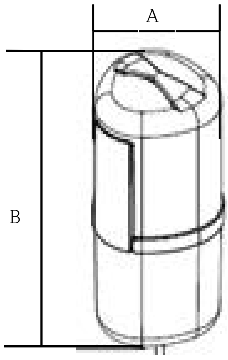

<table>
<tr><th></th><th>100 mL</th></tr>
<tr><td>A</td><td>60.3</td></tr>
<tr><td>B</td><td>125</td></tr>
</table></td></tr>
<tr><td>2-2</td><td>Inner housing2</td></tr>
<tr><td>3-2</td><td>Bottom housing2</td></tr>
<tr><td>1-3</td><td rowspan="3">Light</td><td>Outer housing3</td><td rowspan="3">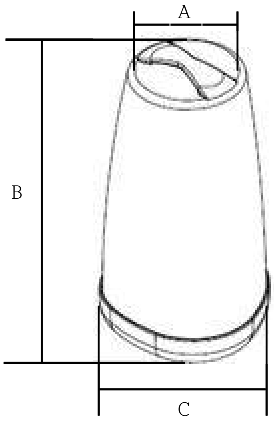

<table>
<tr><th></th><th>100 mL</th></tr>
<tr><td>A</td><td>48</td></tr>
<tr><td>B</td><td>125</td></tr>
<tr><td>C</td><td>68.66</td></tr>
</table></td></tr>
<tr><td>2-2</td><td>Inner housing2</td></tr>
<tr><td>3-1</td><td>Bottom housing1</td></tr>
</table>

<table>
<tr><td>1-4</td><td rowspan="3">Petite</td><td>Outer housing4</td><td rowspan="3">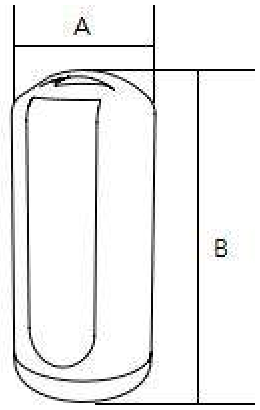

<table><tr><td></td><td>100 mL</td></tr><tr><td>A</td><td>53.2</td></tr><tr><td>B</td><td>116</td></tr></table></td></tr>
<tr><td>2-2</td><td>Inner housing2</td></tr>
<tr><td>3-3</td><td>Bottom housing3</td></tr>
<tr><td>2-1</td><td colspan="2">Housing Silicone Guide</td><td>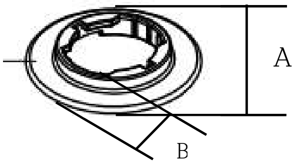

<table><tr><td>A</td><td>45.0</td></tr><tr><td>B</td><td>10.0</td></tr></table></td></tr>
<tr><td>4</td><td colspan="2">T injection port</td><td>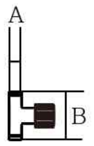

<table><tr><td>A</td><td>6.2</td></tr><tr><td>B</td><td>26.65</td></tr></table></td></tr>
<tr><td>5</td><td colspan="2">I-connector</td><td>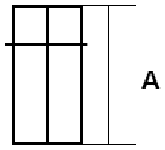

<table><tr><td>A</td><td>15.6</td></tr></table></td></tr>
<tr><td>6-1</td><td colspan="2">Connector Tube(PVC)</td><td>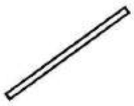
<table><tr><td>O.D</td><td>2.7</td></tr><tr><td>I.D</td><td>1.1</td></tr></table></td></tr>
<tr><td>6-2</td><td colspan="2">Connector Tube(PU)</td><td>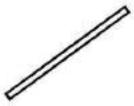
<table><tr><td>O.D</td><td>2.7</td></tr><tr><td>I.D</td><td>1.1</td></tr></table></td></tr>
</table>

<table>
<tr><td>7-1</td><td>Precision flow controller (Selector)</td><td>
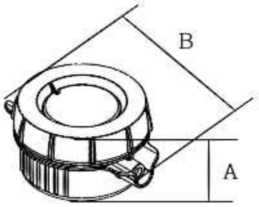

<table><tr><td>A</td><td>25.5</td></tr><tr><td>B</td><td>55.32</td></tr></table>
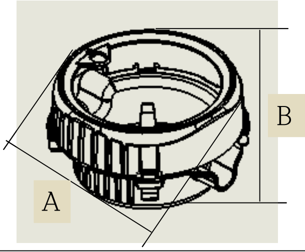

<table><tr><td>A</td><td>45.0</td></tr><tr><td>B</td><td>27.0</td></tr></table>
</td></tr>
<tr><td>7-2</td><td>Precision flow controller (Selector_M)</td><td>
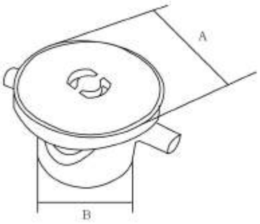

<table><tr><td>A</td><td>32.11</td></tr><tr><td>B</td><td>24.98</td></tr></table>
</td></tr>
<tr><td>8-1</td><td>PCM (Bolus)</td><td>
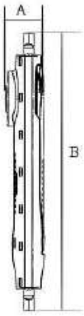

<table><tr><td>A</td><td>17.05</td></tr><tr><td>B</td><td>130.60</td></tr></table>
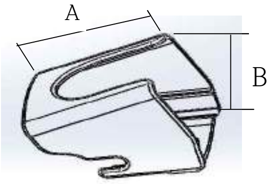

<table><tr><td>A</td><td>35.0</td></tr><tr><td>B</td><td>32.0</td></tr></table>
</td></tr>
<tr><td>8-2</td><td>PCM (Bolus_F)</td><td>
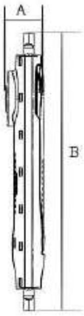

<table><tr><td>A</td><td>14.56</td></tr><tr><td>B</td><td>130.60</td></tr></table>
</td></tr>
<tr><td>9-1</td><td>Filter 1</td><td></td></tr>
</table>

<table>
<tr><td></td><td></td><td>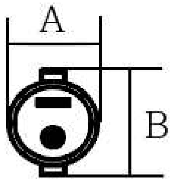

<table><tr><td>A</td><td>20.10</td></tr><tr><td>B</td><td>38.30</td></tr></table></td></tr>
<tr><td>9-2</td><td>Filter 2</td><td>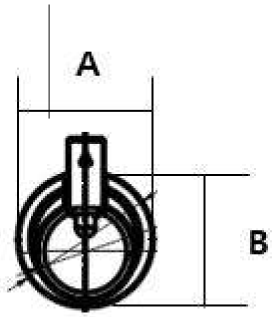

<table><tr><td>A</td><td>21.4</td></tr><tr><td>B</td><td>27</td></tr></table></td></tr>
<tr><td>10</td><td>Slide clamp</td><td>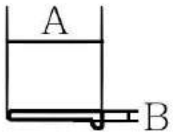

<table><tr><td>A</td><td>25.30</td></tr><tr><td>B</td><td>3.3</td></tr></table></td></tr>
<tr><td>11</td><td>Rotating Male Luer</td><td>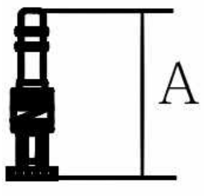

<table><tr><td>A</td><td>45.0</td></tr></table></td></tr>
<tr><td>12</td><td>2-Way Connector</td><td>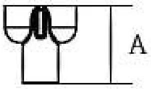

<table><tr><td>A</td><td>15.3</td></tr></table></td></tr>
<tr><td>13</td><td>Restrictor Connector</td><td>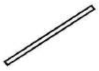
<table><tr><td>O.D</td><td>3.8</td></tr><tr><td>I.D</td><td>2.7</td></tr></table></td></tr>
</table>
